# Adaptive Immunity Induces Tolerance to Flagellin by Attenuating TLR5 and NLRC4-Mediated Innate Immune Responses

**DOI:** 10.3389/fcimb.2019.00029

**Published:** 2019-02-19

**Authors:** Beng San Yeoh, Andrew T. Gewirtz, Matam Vijay-Kumar

**Affiliations:** ^1^Graduate Program in Immunology and Infectious Disease, Pennsylvania State University, University Park, PA, United States; ^2^Center for Inflammation, Immunity and Infection, Institute for Biomedical Sciences, Georgia State University, Atlanta, GA, United States; ^3^Department of Physiology and Pharmacology, University of Toledo College of Medicine and Life Sciences, Toledo, OH, United States; ^4^Department of Medical Microbiology and Immunology, University of Toledo College of Medicine and Life Sciences, Toledo, OH, United States

**Keywords:** LPS, innate immunity, immune tolerance, antibodies, *salmonella*, infection

## Abstract

The host immune system is constantly exposed to diverse microbial ligands, including flagellin (FliC; a ligand for TLR5 and NLRC4) and lipopolysaccharide (LPS; a ligand for TLR4), which could induce immune tolerance to subsequent exposure. Herein, we investigated the extent to which FliC induces self-tolerance *in vivo* and the role of adaptive immunity in mediating such effect. Mice pre-treated with FliC displayed attenuated serum keratinocyte-derived chemokine (KC), interleukin (IL)-6 and IL-18 responses to secondary challenge of FliC. A negative correlation was observed between high anti-FliC titer and reduced KC, IL-6, and IL-18 responses upon FliC re-challenge in WT mice, but not *Rag1*KO mice, suggesting that adaptive immunity could tolerize TLR5 and NLRC4. However, administration of LPS during FliC pre-treatment impaired the generation of anti-FliC antibodies and resulted in a partial loss of self-tolerance to FliC re-challenge. These findings may be relevant in the context of bacterial infection, as we observed that anti-FliC response are protective against systemic infection by *Salmonella typhimurium*. Taken together, our study delineates a distinct co-operative and reciprocal interaction between the innate and adaptive arms of immunity in modulating their responses to a bacterial protein.

## Introduction

The microbial biomass in the intestine is enriched with diverse microbial-associated molecular patterns (MAMPs) that are recognizable by host pathogen recognition receptors (PRRs). Upon activation, PRRs such as Toll-like receptors (TLR) initiate rapid secretion of cytokines and chemokines to recruit immune cells to the site of microbial transgression. To deter unwarranted inflammatory responses, the host has evolved built-in mechanisms to promote immunotolerance against repeated exposure to MAMPs (Rakoff-Nahoum et al., 2004). These mechanisms include strategic expression of PRRs (e.g., TLR5) on the basolateral side of the epithelia (Gewirtz et al., 2001a), downregulation of surface PRRs (e.g., TLR2, TLR4, MD-2, TLR5) (Medvedev et al., 2000; Abreu et al., 2001; Otte et al., 2004), and secretion of endogenous inhibitors [e.g., soluble CD14, secretory IL-1 receptor antagonist (sIL1Ra), soluble TNF receptor I and II] (Liew et al., 2005). Flagelin [FliC; a ligand for TLR5 (Yoon et al., 2012) and NAIP5-NLRC4 (Tenthorey et al., 2017)] is the monomeric protein constituent of flagella, a whip-like appendage, which provides motility for bacteria. As one of the few protein ligand for TLRs, FliC is exceptional in activating both innate and adaptive immunity. Studies have documented that FliC can function as vaccine adjuvant (Mizel and Bates, 2010) and serve as a major antigen in Crohn's disease (Lodes et al., 2004). However, little is known with respect to the functional properties of anti-FliC antibodies (Ab) and whether they could modulate TLR5 response. Lipopolysaccharide [LPS; a TLR4 ligand] is a bacterial glycolipid that comprises the outer membrane of Gram-negative bacteria. Pre-exposure of macrophages and epithelial cells to FliC or LPS, has been reported to induce hyporesponsiveness to subsequent exposures *in vitro* (Medvedev et al., 2000; Otte et al., 2004; Sun et al., 2007). LPS pre-treatment could also regulate TLR5 responses to FliC *in vitro*, though the magnitude of such “cross-tolerance” depends on the doses and cell-type studied (Mizel and Snipes, 2002; Sun et al., 2007; Li et al., 2012).

The purpose of this study was to determine if pre-treating mice with FliC would blunt the innate immune response to FliC re-challenge. We measured serum keratinocyte-derived chemokine (KC) and interleukin (IL)-6 as putative markers for TLR activation. NAIP5-NLRC4 is a cytosolic receptor for FliC, whose activation induces inflammasome; accordingly, we measured serum IL-18 as the marker for its activation. Additionally, we measured the levels of anti-FliC Ab and investigated whether they have a role in dampening the innate immune response upon subsequent FliC re-challenge. We extend this study also to examine the efficacy of anti-FliC Ab against enteric and systemic infection by *Salmonella typhimurium*.

## Materials and Methods

### Reagents

FliC from *S. typhimurium* (SL3201, fljB-) was purified through sequential cation and anion-exchange chromatography as previously described (Gewirtz et al., 2001b). LPS from *Escherichia coli* 0128:B12 was purchased from Sigma-Aldrich (St. Louis, MO). Reagents and antibodies for ELISA were purchased from R&D Systems (Minneapolis, MN).

### Mice

C57BL/6 WT and *Rag1*KO mice were procured from Jackson Laboratories (Bar Harbor, ME) and bred under specific pathogen-free condition at Pennsylvania State University (PSU). Mice were housed in cages containing corncob bedding and nestlet, fed *ad libitum* and maintained at 23°C with a 12 h light/dark phase cycle. Animal experiments were approved by the Institutional Animal Care and Use Committee (IACUC) at PSU.

### Flagellin and LPS Challenge

Six-weeks-old male WT mice (*n* = 4) were treated with PBS, LPS (10 μg), FliC (50 μg), LPS→ FliC, and FliC→ LPS (arrow denoting secondary challenge 3 h after the initial treatment). On day 18, mice were bled minimally and assayed for seroreactivity to FliC and LPS. On day 21, mice were re-challenged with FliC (50 μg) and bled after 2 h. In another experiment, six-weeks-old male *Rag1*KO mice (*n* = 4) were treated with PBS or FliC at day 0 and re-challenged with FliC at day 21.

### Serum Collection

Blood was collected into BD microtainer (BD Biosciences, San Jose, CA) via non-terminal retro-orbital bleeding. Hemolysis-free sera were obtained after centrifugation and stored at −80°C until further analysis.

### Cell Culture

Human colon adenocarcinoma cell line HT29 was cultured on 24-well plates in Dulbecco's modified Eagle medium (DMEM) supplemented with 10% fetal bovine serum (FBS) and 1% penicillin and streptomycin. After cells became confluent, the media was replaced with incomplete DMEM without FBS. Cells were co-treated with PBS or FliC (50 ng/mL) in combination with serum (1:50 dilution) from FliC-treated WT, naïve WT, or FliC-treated *Rag1*KO mice. Cells were incubated for 5 h at 37°C and 5% CO_2_. Culture supernatant was collected and stored at −80°C until further analysis.

### Enzyme-Linked Immunosorbent Assay (ELISA)

Mouse KC and IL-6, and human IL-8 were analyzed using ELISA kits (R&D Systems) according to the manufacturer's instructions. Mouse IL-18 were analyzed via an in-house ELISA as described previously (Zhang et al., 2014). To measure IgG seroreactivity to FliC and LPS, sera were diluted (1:500) and analyzed via ELISA as described previously (Ziegler et al., 2008).

### Bacterial Culture

*Salmonella enterica* subsp. *enterica serovar* Typhimurium (strain SL3201) were cultured for 15 h in Luria-Bertani (LB) broth at 37°C with shaking (200 rpm). Bacteria CFU were adjusted based on OD at 600 nm.

### Murine Models of *Salmonella* Infection

Six-weeks-old male WT mice were administered PBS or FliC (50 μg; i.p.) at day 0 and 14. On day 21, mice were infected with *Salmonella* either orally (1 × 10^8^ CFU; *n* = 5) or via i.p. (1 × 10^4^ CFU; *n* = 10) as previously described (Vijay-Kumar et al., 2008a). Mice were monitored for body weight loss and mortality.

### Statistical Analysis

Values are presented as mean ± SEM. Data were analyzed using one-way analysis of variance (ANOVA) followed by *post-hoc* Tukey's multiple comparison test. Spearman correlation was used to establish the association of two variables. Kaplan–Meier survival curves were analyzed with log-rank (Mantel-Cox) test. Data were considered significant at *p* < 0.05. All analyses were performed using GraphPad Prism 6.0 (La Jolla, CA).

## Results

### LPS Suppresses the Adaptive Immune Response to Flic *in vivo*

**Figure 1:**
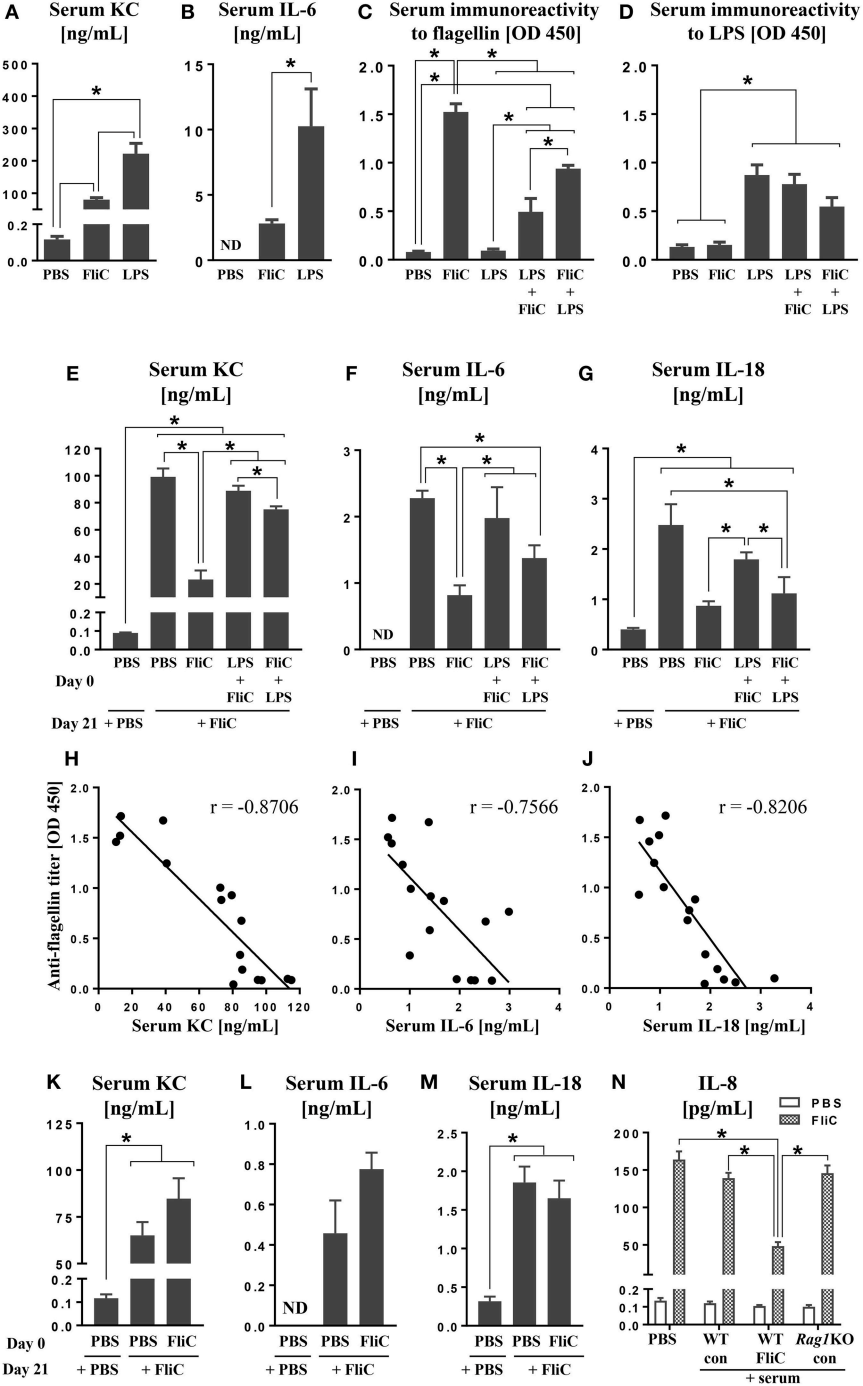
Anti-FliC Ab attenuates TLR5 and NAIP5-NLRC4 cytokine responses to FliC re-challenge. Six week old male WT mice (*n* = 4) were challenged with either PBS, LPS (10 μg) or FliC (50 μg). Sera were collected after 2 h and analyzed for **(A)** KC and **(B)** IL-6. In another experiment, 6 week old WT mice (*n* = 4) were challenged with PBS, LPS, FliC, LPS→ FliC, FliC→ LPS with the arrow denoting secondary challenge 3 h after the initial treatment. At day 18 post-treatment, sera were collected and measured for **(C)** seroreactivity to flagellin and **(D)** seroreactivity to LPS. At day 21 post-treatment, the mice were re-challenged with FliC (50 μg). Sera were collected after 2 h and analyzed for **(E)** KC, **(F)** IL-6, and **(G)** IL-18. Correlations between seroreactivity to FliC against serum **(H)** KC [Spearman correlation (*r* = −0.8706; *p* (two-tailed) < 0.0001)], **(I)** IL-6 (*r* = −0.7566; *p* = 0.0007), **(J)** IL-18 (*r* = −0.8206; *p* = 0.0002). Six week old male *Rag1*KO mice (*n* = 4) were pre-treated with either PBS or FliC (50 μg) at day 0. At day 21, mice were challenged with FliC. Sera were collected 2 h post-treatment and analyzed for **(K)** KC, **(L)** IL-6, and **(M)** IL-18. HT29 cells were co-treated with FliC and serum from either FliC-treated WT, naïve WT, or FliC-treated *Rag1*KO mice. **(N)** Five-hour culture supernatants were assayed for secretion of IL-8. Results presented as mean ± SEM and are representative of two independent experiments. ND, not detected. Data in **(H–J)** were analyzed with Spearman correlation test. All other data were analyzed via ANOVA with *post-hoc* Tukey's test with ^*^p < 0.05.

The immune system would be exposed to multiple TLR ligands, particularly during polymicrobial sepsis. While MAMPs have been shown to induce immune tolerance *in vitro* (Medvedev et al., 2000; Mizel and Snipes, 2002; Otte et al., 2004; Sun et al., 2007; Li et al., 2012), the occurrence of tolerance is relatively under-studied *in vivo* (De Vos et al., 2009). First, we estimated the cytokine responses in mice challenged with approximately equimolar concentration of 10 μg LPS (~10 kDa) or 50 μg FliC (~50 kDa). LPS-treated mice displayed ~ 2-fold more increase in serum KC and IL-6 than FliC-treated mice (Figures 1A,B). Such observation is consistent with our previous study demonstrating that LPS is more potent in inducing innate immunity, including TNFα response, than FliC (Vijay-Kumar et al., 2008b).

Studies from our group (Sanders et al., 2006, 2008, 2009; Vijay-Kumar et al., 2010) and others (Letran et al., 2011; Atif et al., 2014; Lopez-Yglesias et al., 2014; Li et al., 2016) demonstrate that TLR5 is required for promoting optimal adaptive responses to FliC. We asked whether such response could be impeded when mice were administered with LPS shortly before or after FliC. Assessment on mice at day 18 post-treatment revealed that their seroreactivity to FliC was in the order of FliC > FliC→ LPS > LPS→ FliC > PBS (Figure 1C). Increased seroreactivity to LPS was also observed in the order of LPS ≥ LPS→ FliC ≥ FliC→ LPS (Figure 1D), albeit not reaching significance, but nonetheless suggest that anti-LPS response could also be disrupted by FliC. The negligible levels of anti-FliC Ab in mice given only LPS, as well as anti-LPS Ab in mice given only FliC, indicated the specificity of the Ab generated (Figures 1C,D).

### Anti-FliC Antibodies Dampen the Innate Immune Response to FliC

Next, we investigated whether anti-FliC could modulate TLR5 responses. To address this possibility, we re-challenged the aforementioned FliC/LPS-treated mice on day 21 with FliC. Serum KC, IL-6, and IL-18 induced by FliC were strikingly diminished in FliC pre-treated mice, when compared to naïve mice given FliC for the first time (Figures 1E–G). Mice pre-treated with LPS→ FliC or FliC→ LPS also displayed modest reduction in serum KC, IL-6, and IL-18 following FliC re-challenge (Figures 1E–G). Regression analyses affirmed the strong negative correlations between serum anti-FliC Ab with either KC, IL-6, or IL-18 levels (Figures 1H–J), thus implicating the involvement of adaptive immunity underlying the tolerance to FliC.

### Loss of Adaptive Immunity Abrogates Immune Tolerance to FliC Re-challenge

To address whether adaptive immunity is required for the tolerance to FliC, we employed RAG1-deficient (*Rag1*KO) mice, which lack mature T and B cells and, therefore, are unable to generate any Ab. Unlike WT mice, *Rag1*KO mice were not refractory to FliC re-challenge (Figures 1K–M) regardless of whether they have or have not been previously exposed to FliC. Such outcomes imply that the tolerance observed in WT mice is likely antibody-dependent. To affirm such notion, we co-treated HT29 cell line (a model intestinal epithelia) with FliC and serum from FliC-treated or untreated WT mice; FliC-treated *Rag1*KO mice sera was used as negative control. As anticipated, only serum with anti-FliC suppressed FliC-induced secretion of IL-8 (human homolog of KC) from HT29 cells (Figure 1N).

### Adaptive Response to FliC Protects Mice Against *Salmonella* Infection

**Figure 2:**
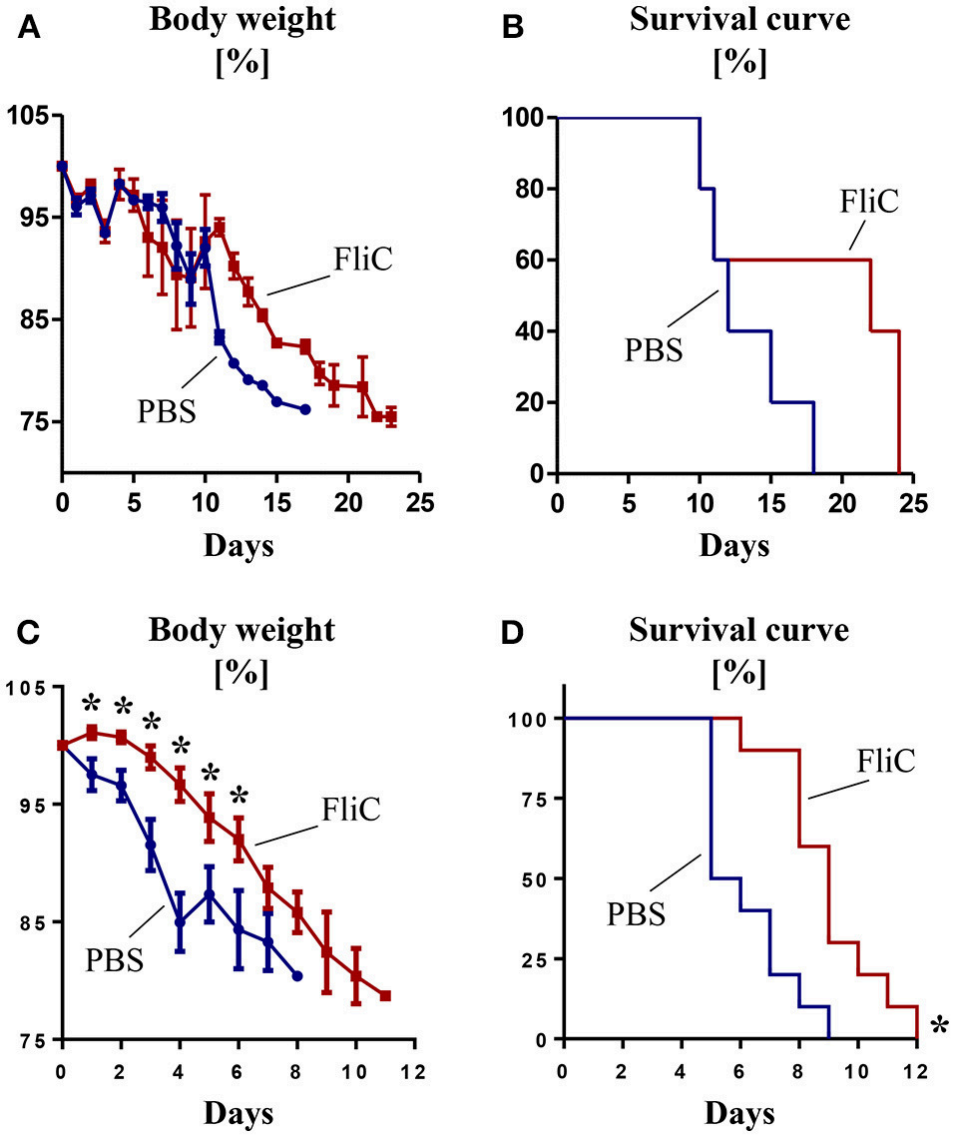
Adaptive immunity to FliC protects mice from *Salmonella* infection. Six week old male WT mice were treated with FliC (50 μg) on day 0 and 14, and infected with *Salmonella typhimurium* on day 21. **(A)** Percent body weight and **(B)** Kaplan–Meier survival curve for mice receiving oral infection (10^8^ CFU bacteria; *n* = 10). **(C)** Percent body weight and **(D)** Kaplan-Meier survival curve for mice receiving intraperitoneal infection (10^4^ CFU bacteria; *n* = 10). Results presented as mean ± SEM. Data **(A–D)** are pooled from two independent experiments. ^*^p < 0.05 (Student's *t* test) for **(A,C)**; ^*^p < 0.005 [Log-rank (Mantel-Cox) test] for **(B,D)**.

Having demonstrated the role of anti-FliC in mitigating TLR5 response, we next investigated their physiological significance in a natural infection model of *S. typhimurium*. Compared to controls, mice pre-treated with FliC displayed a slight trend toward a delayed body weight loss and mortality upon oral infection (Figures 2A,B). Such protection was more pronounced when *Salmonella* was administered intraperitoneally to mimic systemic infection (Figures 2C,D). These findings suggest that adaptive immunity to FliC are not bystander responses, but may have a role in conferring protection against enteric and systemic infection by *Salmonella*.

## Discussions

Here we delineate a reciprocal interaction between the innate and adaptive immunity in modulating host responses to FliC *in vivo*. Our results demonstrate that anti-FliC Ab are capable of exerting tolerance to subsequent FliC re-challenge. The attenuation in cytokine responses downstream of TLR5 and NLRC4 could be, in part, due to formation of FliC-Ab complex that may undergo rapid degradation via Fc receptor-mediated phagocytosis. While the binding sites between TLR5 and FliC have been elucidated (Smith et al., 2003), the antibody binding sites to FliC is not clear. Further studies are required to interrogate the antibody isotype variants of anti-FliC Ab, as well as the epitope and paratope interaction between FliC and anti-FliC, respectively. Deciphering such molecular interactions may help in designing anti-FliC Ab with high affinity and avidity for passive immunization to treat flagellated bacterial infections.

Intriguingly, administration of LPS during the initial FliC challenge interfered with the anti-FliC response. As TLR4 and TLR5 share a common signaling pathway (i.e., MyD88) (Mizel and Snipes, 2002; Sato et al., 2002; Otte et al., 2004), it is possible that activation of the former by LPS could have dampened the latter. Such cross-tolerance may explain the suboptimal anti-FliC Ab response that requires proper TLR5 function. LPS could also disrupt proper antigen processing or presentation of FliC, potentially via inducing proteases that degrade FliC or serving as a competing antigen. Despite LPS being regarded as a potent vaccine adjuvant (Coffman et al., 2010), it is interesting to note that it has poor adjuvanicity in potentiating anti-FliC Ab generation.

Though we observed modest improvement in overall survival in FliC-treated mice over naïve mice against *Salmonella* infection, the extent of protection appears to depend on the route and course of infection. Since oral infection requires the pathogen to first colonize and breach the mucosal barrier, this would provide opportunity for *Salmonella* to alter FliC expression and thus evade the host innate immune system. Interaction with the host cell membrane lysophospholipid cues *Salmonella* to secrete FliC monomers (Subramanian and Qadri, 2006), thus activating innate immunity (Gewirtz et al., 2001b) and recruiting macrophages to serve as carriers for dissemination. Upon transitioning from extra- to intra-cellular phase of infection, *Salmonella* could downregulate their FliC expression (Cummings et al., 2005, 2006; Alaniz et al., 2006). On the flip side, administration of *Salmonella* directly into systemic circulation renders them more susceptible to anti-FliC Ab since the pathogen may not sufficiently and timely downregulates FliC expression.

Although anti-FliC Ab are integral for immunity against *Salmonella*, their functional capacities are poorly characterized. It was not until much recently that anti-FliC Ab are shown not to be mere bystanders, but could mediate anti-bacterial activity in humans (Maclennan et al., 2008; Ramachandran et al., 2016). Our present study extends this body of knowledge by elucidating that anti-FliC Ab also counter-regulates innate immunity following repeated exposure to FliC and that this regulation could be hindered by LPS.

## Author Contributions

BY performed mouse experiments, *in vitro* assays and analyzed the results. AG and MV-K assisted with data analysis and provided critical discussions. BY and MV-K wrote the manuscript.

### Conflict of Interest Statement

The authors declare that the research was conducted in the absence of any commercial or financial relationships that could be construed as a potential conflict of interest.

## Abbreviations

Ab: antibodies
FliC: flagellin
IL: interleukin
KC: keratinocyte-derived chemokine
LPS: lipopolysaccharide
MAMPs: microbial-associated molecular patterns
PRRs: pathogen recognition receptors
TLR: Toll-like receptors.
